# Supplementary material for: From sequence to enzyme mechanism using multi-label machine learning
Source: BMC Bioinformatics. 2014 May 19;15:150. doi: 10.1186/1471-2105-15-150 (PMC4229970; doi:10.1186/1471-2105-15-150)
Supplement: Additional file 2 — Java code of ml2db. Additional file ml2db_code.tar.gz contains the Java source code to run the multi-label machine learning experiments and save the results to database. The code’s Javadoc is included. [file 1471-2105-15-150-S2.zip › additional file 2/ml2db/ml2db/doc/mulan/data/ConverterLibSVM.html]

ConverterLibSVM


JavaScript is disabled on your browser.


- Overview
- Package
- Class
- Use
- Tree
- Deprecated
- Index
- Help

- Prev Class
- Next Class

- Frames
- No Frames

- All Classes

- Summary:
- Nested |
- Field |
- Constr |
- Method

- Detail:
- Field |
- Constr |
- Method


mulan.data

## Class ConverterLibSVM

- java.lang.Object
- - mulan.data.ConverterLibSVM

- ---

    

  ```
  public class ConverterLibSVM
  extends java.lang.Object
  ```

  Class that converts LibSVM multi-label data sets to Mulan compatible format

  Version:
  :   $Revision: 1.1 $

  Author:
  :   Grigorios Tsoumakas

- - ### Constructor Summary

    Constructors

    Constructor and Description

    `ConverterLibSVM()`
  - ### Method Summary

    Methods

    | Modifier and Type | Method and Description |
    | --- | --- |
    | `static void` | `convertFromLibSVM(java.lang.String path, java.lang.String sourceFilename, java.lang.String targetFilestem, java.lang.String relationName)` Converts a multi-label dataset from LibSVM format to the format that is compatible with Mulan. |
    | `static void` | `main(java.lang.String[] args)` Command line interface for the converter |

    - ### Methods inherited from class java.lang.Object

      `equals, getClass, hashCode, notify, notifyAll, toString, wait, wait, wait`

- - ### Constructor Detail


    - #### ConverterLibSVM

      ```
      public ConverterLibSVM()
      ```
  - ### Method Detail


    - #### convertFromLibSVM

      ```
      public static void convertFromLibSVM(java.lang.String path,
                           java.lang.String sourceFilename,
                           java.lang.String targetFilestem,
                           java.lang.String relationName)
      ```

      Converts a multi-label dataset from LibSVM format to the format
      that is compatible with Mulan. It constructs one ARFF and one XML file.

      Parameters:
      :   `path` - the directory that contains the source file and will contain
          the target files
      :   `sourceFilename` - the name of the source file
      :   `relationName` - the relation name of the arff file that will be
          constructed
      :   `targetFilestem` - the filestem for the target files (.arff and .xml)


    - #### main

      ```
      public static void main(java.lang.String[] args)
      ```

      Command line interface for the converter

      Parameters:
      :   `args` - command line arguments


- Overview
- Package
- Class
- Use
- Tree
- Deprecated
- Index
- Help

- Prev Class
- Next Class

- Frames
- No Frames

- All Classes

- Summary:
- Nested |
- Field |
- Constr |
- Method

- Detail:
- Field |
- Constr |
- Method
